# Different application methods of humic acid and zinc differentially regulate osmotic balance and C-repeat binding factor pathways in cold-stressed oat (*Avena sativa* L.)

**DOI:** 10.7717/peerj.20927

**Published:** 2026-03-06

**Authors:** Imren Kutlu, Nurdilek Gulmezoglu, Tevrican Dokuyucu

**Affiliations:** 1 Field Crops, Osmangazi University, Eskisehir, Turkey; 2 Soil Science and Plant Nutrition Department, Agricultural Faculty, Osmangazi University, Eskisehir, Turkey; 3 Field Crops Department, Agricultural Faculty, Kahramanmaras Sütcü Imam University, Kahramanmaras, Turkey

**Keywords:** Turgor loss, Leaf water content, Proline accumulation, CBF genes, Zinc, Humic acid, Seed priming, Cold tolerance

## Abstract

The present study investigated the effects of different humic acid (HA) and zinc (Zn) application methods on membrane durability index (MDI), leaf relative water content (RWC), turgor loss (TL), proline content (PC), and C-repeat binding factor (CBF) gene expressions in oat plants exposed to various low temperatures. For this purpose, two oat cultivars—Albatros (cold-sensitive) and Checota (cold tolerant)—were grown under controlled conditions with HA and Zn applied to the seeds or soil, either individually or in combination, until the 3–4 leaf stage. The plants were subsequently exposed to temperatures of 4 °C, 0 °C, −5 °C, −10 °C, and −15 °C for 24 h each. The results indicated that the application methods of HA and Zn substantially influenced the plants’ responses to low temperature. Among the treatments, soil application of HA+Zn (SA_HA+Zn), seed priming with HA combined with soil-applied Zn (SP_HA+SA_Zn), and seed priming with Zn combined with soil-applied HA (SP_Zn+SA_HA) provided the greatest protection against cold stress, as evidenced by improved MDI, RWC, TL, and PC levels. Gene expression analyses further revealed that low temperatures upregulated the CBF genes and the related regulatory genes VRN1 and ZAT12, with the strongest induction observed under SA_HA+Zn, suggesting that this combined approach more effectively activates the plant’s cold defense mechanisms.

## Introduction

The oat (*Avena sativa* L.) stands out as one of the principal cereals utilized for both human nutrition and as livestock feed, forage, hay, and silage production, occupying approximately 20 million hectares of cultivated land worldwide (Tomar & Singh, 2024). Oat demonstrates extensive ecological plasticity, enabling it to thrive across a range of soil types and to outperform other cereals such as wheat and maize even in nutrient-poor soils. Moreover, in forage-based systems it is often used as an alternative or complement to silage maize and alfalfa due to its adaptability to cooler climates and marginal soils (Ye et al., 2023). However, oats are susceptible to environmental stresses, including high temperatures, drought, and freezing conditions (Nokhsorov et al., 2024). Exposure to low temperatures is a significant limiting factor that profoundly influences the geographical distribution of crops.

The principal physiological processes plants use to mitigate the negative effects of cold stress are maintaining membrane stability and regulating cellular osmotic balance. Due to its heightened sensitivity to temperature fluctuations, the plasma membrane is used to maintain membrane fluidity and prevent phase transitions that can lead to rigidity under cold stress (Sardoei & Fazeli-Nasab, 2025). Extracellular ice formation is a common occurrence when temperatures drop below freezing, leading to water extraction from cells and inducing osmotic stress. This process can lead to membrane rupture and subsequent cellular dehydration (Wang et al., 2024). Plants acclimated to low temperatures have been observed to accumulate proline and other osmolytes, which help ameliorate the adverse effects of low temperature stress. These solutes help stabilize macromolecules and lessen the chance of intracellular ice formation. The combination of these physiological adaptations supports metabolic activity and membrane integrity in freezing temperatures (Zhou et al., 2025).

Plants activate the ICE1-CBF-COR transcriptional cascade as a key mechanism for cold acclimation at the molecular level. In this pathway, C-repeat binding factors (CBFs) from the AP2/ERF family bind CRT/DREB elements in the promoters of cold-regulated (COR) genes and coordinate a complex physiological response that includes osmolyte biosynthesis, reactive oxygen species (ROS) detoxification, and hormone metabolism (Jan et al., 2017; Shi, Ding & Yang, 2018; Qian, He & Li, 2024). Four specific CBF/DREB genes in oats—AsCBF1, AsCBF2, AsCBF3, and AsCBF4—have different roles in establishing cold tolerance further regulated by zinc finger protein ZAT12 and frost-tolerance marker AsVRN1 (Fowler, Cook & Thomashow, 2005; Bräutigam, 2009).

Genotype primarily controls these molecular changes, but external applications of humic acid (HA) and zinc (Zn) significantly alter the transcriptional and physiological frameworks of stress resistance. HA is a biochemical trigger that regulates developmental programs by interacting with protein phosphorylation pathways to activate stress-responsive transcription factors (Trevisan et al., 2011; Canellas et al., 2024). Zn complements these alterations by ensuring structural integrity for biological membranes while being indispensable for the functionality of transcription factors with zinc finger domains that are central to plant antioxidant defense (Çakmak et al., 1999; Kaznina et al., 2022).

Despite increasing evidence about the modulation of stress responses by humic substances and micronutrients like Zn, there is a glaring unavailability of research directly linking varied application methods of HA and Zn with the regulation of major cold-responsive transcription factors, especially the CBF regulon in oats. Furthermore, comparative studies under extreme and sequential cold regimes have been scanty. Such knowledge gaps warrant an urgent need to study how biostimulant-micronutrient interactions can affect particular pathways to cold tolerance. Therefore, this study assessed the effects of HA and Zn applied *via* different methods on osmotic status as well as expression profiles for AsCBFs, AsVRN1, and AsZAT12 genes in oat plants under cold stress. The objective of this study was twofold: (I) to elucidate the role of HA and Zn in cold tolerance in plants, and (II) to determine the most suitable application method in practice.

## Materials and Methods

### The plant, soil and application materials

In this study, winter (cv. Checota)- and spring (cv. Albatros) oat (*Avena sativa* L.) varieties were used as the plant materials. The plants were grown in soil obtained from Eskişehir-Sultanönü region, as it has low Zn and organic matter content. In plant-growing soils, Zn sufficiency is generally defined as DTPA-extractable Zn concentrations above 0.5–1.0 mg kg^−1^, whereas lower levels are considered deficient for most crops (Alloway, 2008). The properties of the soil and HA material used are given in Table 1. The soil is slightly alkaline and sandy loam in structure. The salt content is low, and the copper and lime content are sufficient. The levels of organic matter, potassium, phosphorus, manganese, zinc, and iron are found to be inadequate. HA material (TKI-HUMAS) is a leonardite-derived product with a high humic and fulvic acid (FA) content. Additionally, zinc sulfate heptahydrate (ZnSO_4_.7H_2_O) with a purity of 99%, containing 22.6% elemental Zn (Catalog No. 1.08883; Merck, Darmstadt, Germany), was used as a zinc source.

**Table 1:** The properties of the experimental soil and humic acid material.

	Soil	Humic acid
pH	7.67	11.8
EC (dS m^−1^)	1.20	5.6
Lime (%)	11.83	---
Organic matter (%)	0.65	5.0
Texture	Sandy loam	---
Humic+Fulvic acid (%)	---	12.0
Total N (%)	0.05	0.23
P (mg kg^−1^)	0.49	72.0
K (mg kg^−1^)	136	16,000
Ca (mg kg^-1^)	2,100	5,228
Mg (mg kg^−1^)	450	680
Fe (mg kg^−1^)	1.20	550
Cu (mg kg^−1^)	1.10	2.4
Mn (mg kg^−1^)	0.89	6.2
Zn (mg kg^−1^)	0.23	2.4
Na (mg kg^−1^)	19.0	4,900

### Experimental setup

The experiment was designed as a factorial experimental design with three replicates, six temperature level, eight HA and Zn treatments and two oat cultivars. Oat plants were grown in plastic pots (20.3 cm diameter × 17 cm height), each containing four kg of growth medium. To minimize positional effects, pot positions were randomized at the beginning of the experiment and maintained under uniform light and temperature conditions throughout the growth period. The plant growing medium was composed of a 1:1 ratio of soil and perlite. The basal fertilization was applied at rates of 200 mg N kg^−1^ soil as (NH_4_)_2_SO_4_, 100 mg P kg^−1^ soil and 125 mg K kg^−1^ soil as KH_2_PO_4_, together with 2.5 mg Fe kg^−1^ soil supplied as Fe-EDTA. Following these procedures, all pots were watered with pure water until they reached field capacity (70%).

The HA and Zn treatments, applied individually and in combination, were included to assess both their independent and potential interactive effects on cold stress tolerance in oat. The HA and Zn treatments included in the experiment were carried out as described below.

1. Control (No_HA/No_Zn) refers to plants that did not receive any HA or Zn application.
2. Seed priming HA (SP_HA): The oat seeds were treated with a HA solution prepared according to the manufacturer’s recommended dose for cereals (2 L HA per 100 kg seed). Accordingly, 0.30 mL of HA was applied per 15 g of seed.
3. Seed priming with Zn (SP_Zn): A 0.3% Zn stock solution was prepared by dissolving 13.194 g of ZnSO_4_·7H_2_O in 1 L of distilled water. Seeds of each oat genotype were placed in 90 mm Petri dishes, treated with 10 mL of the Zn solution, and incubated at 25 °C for 12 h (Johnson et al., 2005). After air-drying, the seeds were transplanted into pots.
4. Soil application HA (SA_HA): In accordance with the manufacturer’s guidelines for the application of HA, 0.024 ml of HA was applied per kg of soil.
5. Soil application Zn (SA_Zn): ZnSO_4_.7H_2_O was applied to the soil at a rate of 5 mg Zn kg^−1^ soil, in combination with the basic fertilization, prior to planting (Kalayci et al., 1999).
6. Soil application HA + Zn (SA_HA+Zn): Both HA and Zn were applied to the soil together at the same rates described for SA_HA and SA_Zn.
7. SP_HA+SA_Zn: The oat seeds were primed with HA as described in SP_HA, and Zn was applied to the soil as described in SA_Zn.
8. SP_Zn+SA_HA: Seeds were primed with Zn as described in SP_Zn, and HA was applied to the soil as described in SA_HA.

The plants belonging to each group were cultivated for 28 days until the plants reached the 3–4 leaf stage under controlled conditions (25/16 °C [day/night], 70% humidity, 10 Klux of light, and a 16/8-h photoperiod [day/night]). At the conclusion of the designated period, the non-stressed (25 °C) plants that did not undergo low-temperature treatment were harvested, and samples were collected for subsequent analysis. The temperature of the plant growth chamber was set to +4 °C, and the remaining plants were maintained at this temperature for a 24-h period. Subsequently, the temperature of the plant growth chamber was reduced to 0 °C, −5 °C, −10 °C, and −15 °C, respectively. Samples were collected from the stressed plants at the conclusion of each 24-h period. The cold stress treatments were conducted following the protocol of Kutlu & Turhan (2016) with some modifications.

### Plant analyses

*The determination of the membrane durability index (MDI):* Three 1-cm-long sections were extracted from the harvested leaf samples. These sections were washed with pure water, dried, placed in test tubes, and 20 ml of pure water was added to them. The prepared samples were incubated at room temperature for 4 h in a shaker at 250 rpm. The initial readings were obtained with an EC meter to ascertain the resulting electrolyte leakage (A). Subsequently, the tubes were autoclaved at 121 °C for 15 min, a process that ensures complete tissue death. After the samples reached room temperature, the electrolyte leakage measurement (B) was repeated.

The MDI is calculated using the following formula: MDI = (1 – A/B) × 100

The relative water content (RWC) and turgor loss (LT) of the plants were determined by extracting three 2-cm-long sections from the leaf samples. Fresh weights (FW) were recorded immediately, followed by turgor weights (TW) after 4 h in pure water and dry weights (DW) after 24 h at 70 °C. The “RWC = (FW−DW)/(TW−DW) × 100” formula were used to determine the RWC and the “LT = (TW−FW)/TW × 100” formula for LT values based on the collected data. The results were then reported as a percentage.

Proline analysis was performed as described by Bates, Waldren & Teare (1973). The oat leaves were homogenized with 3% sulfosalicylic acid and centrifuged at 6,000 rpm for 10 min. The resulting supernatant was transferred to test tubes, and glacial acetic acid and acid ninhydrin were added. The samples were then incubated in a 100 °C water bath for 1 h. After cooling, toluene was added, and the samples were taken from the upper phase. The concentrations were determined at 520 nm *via* a spectrophotometer (Thermo-Aquamate; Thermo Fisher Scientific, Waltham, MA, USA) on a calibration curve prepared using pre-prepared proline standards. The proline content was calculated as µmol proline/g fresh weight.

Gene expression analyses for all treatment groups were conducted following a standardized workflow. Total RNA was extracted from 100 mg of finely powdered leaf material flash-frozen in liquid nitrogen using the RNeasy Plant Mini Kit (Qiagen, CA, USA). RNA concentration and purity were determined spectrophotometrically with a NanoDrop instrument (Thermo ND2000; Thermo Fisher Scientific, Waltham, MA, USA). Only samples with an A260/A280 ratio of ≥2.0 were considered suitable for downstream analyses; samples below this threshold were re-isolated to ensure RNA integrity and purity. RNA samples were subsequently adjusted to a uniform concentration of 1,000 ng/µL prior to complementary DNA (cDNA) synthesis. Residual genomic DNA contamination was eliminated using RQ1 RNase-free DNase (Promega). First-strand cDNA synthesis was performed with the VitaScript™ First-Strand cDNA Synthesis Kit (PCCSKU1301; Procomcure Biotech, Thalgau, Austria) in accordance with the manufacturer’s instructions. The synthesized cDNA was then diluted to a working concentration of 100 ng/µL and used as a template for quantitative real-time PCR (RT-qPCR). Amplification reactions were carried out using the 2X Magic SYBR Kit (Procomcure) on a CFX Connect Real-Time PCR Detection System (Bio-Rad, California, USA). The thermal cycling protocol consisted of an initial denaturation at 95 °C for 5 min, followed by 40 cycles of denaturation at 95 °C for 15 s, annealing at 60 °C for 30 s, and extension at 72 °C for 30 s. Relative expression was calculated using the 2^–ΔΔCt^ method (Livak & Schmittgen, 2001). For each cultivar, all HA/Zn treatments were evaluated at each temperature level using three independent biological replicates, resulting in a full factorial experimental design. Gene-specific primers for CBFs, VRN1, ZAT12, and the reference gene AsActin, which were experimentally validated for amplification efficiency and species specificity in previous studies, were employed in this study. Primer sequences and their respective references are summarized in Table 2. Prior to qRT-PCR analysis, the thermodynamic characteristics and specificity of the primers were further re-validated *in silico* using Primer3 software and NCBI Primer-BLAST against the *Avena sativa* genome database to ensure accurate target amplification.

**Table 2:** Primer sequences of the genes subjected to expression analysis.

Target gene	Primer sequences	Reference
AsCBF1	F: CCACAGTCCACCGTATCAGCAAG	Bräutigam et al. (2005)
	R: CGTCTCCTTGAACTTGGTGCG	
AsCBF2	F: CCAATGTCCCAGCAGATGAG	Bräutigam et al. (2005)
	R: CCCAGTTGTTCCATCCATAGAG	
AsCBF3	F: CGGGCAAAGTTGAGGCAGGC	Bräutigam et al. (2005)
	R: TAGGCTCTGGCTCGGCACCTTC	
AsCBF4	F: CCCAGCCTTCAGCAGCGTC	Bräutigam et al. (2005)
	R: TCTCCACAGTCTCCTCCGTGC	
AsVRN1	F: GACTCATCATCTTCTCCACCA	Bräutigam (2009)
	R: TCTGCTTCTCCACGAGTTCC	
ZAT12	F: CAGTTTCATTCGTTCCAAGCCT	Doherty et al. (2009)
	R: TCCACCATCCCTAGACTCAG	
AsActin	F: GCGACAATGGAACTGGC	Bräutigam et al. (2005)
	R: GTGGTGAAGGAGTAACCTCTCTCG	

### Statistical analysis

IBM SPSS 26 was used to perform all statistical procedures and generate the figures. The study’s data were subject to analysis of variance (ANOVA) using a completely randomized factorial design, separately for each HA and Zn treatment. The figures present the data’s mean and standard error (SEM) through the use of bar graphs and error bars.

## Results

### Membrane durability index (MDI) of oat plants under low temperature stress

**Figure 1:**
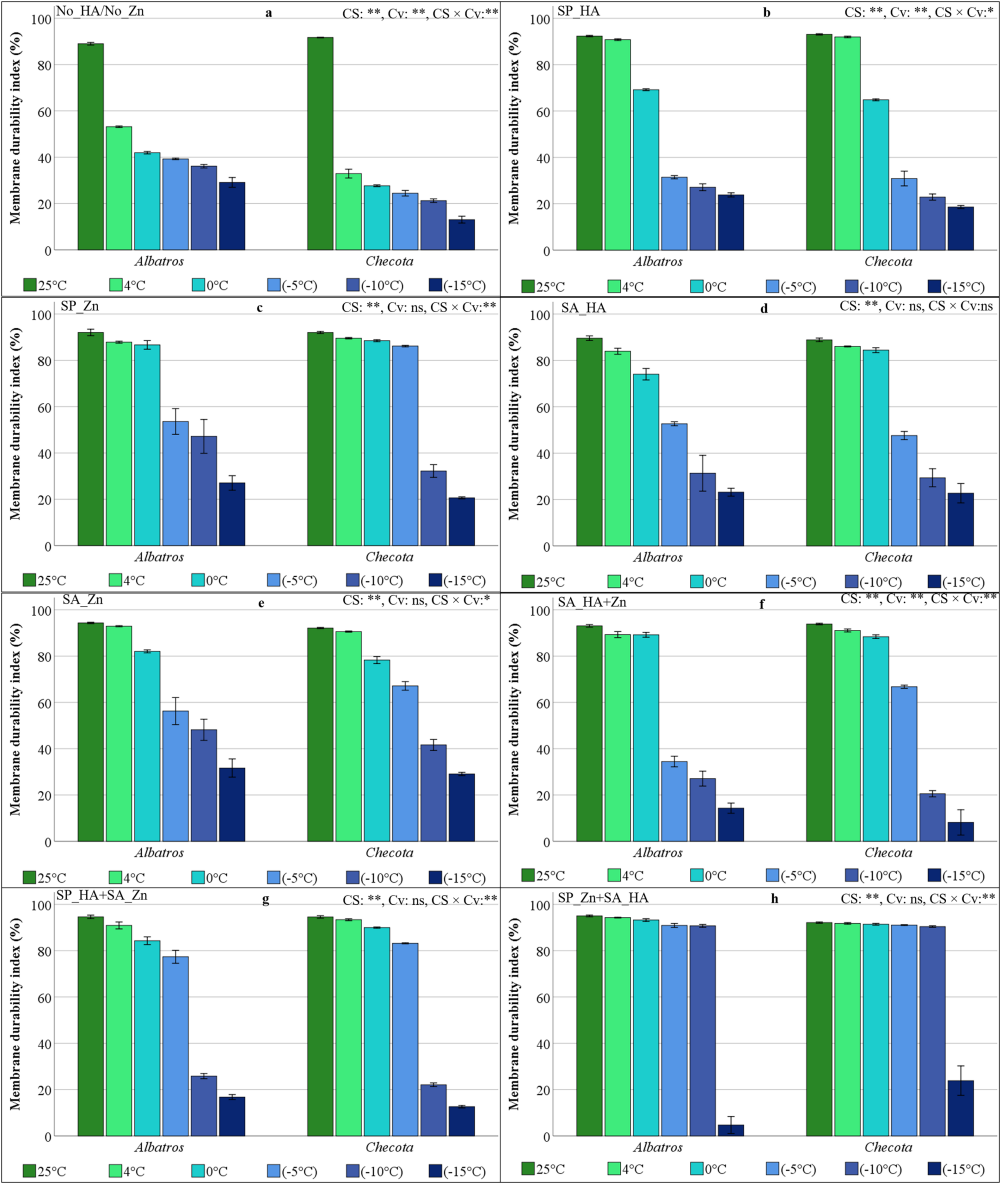
Membrane durability index (%) of two oat cultivars (Albatros and Checota) exposed to decreasing temperatures (25 °C, 4 °C, 0 °C, −5 °C, −10 °C and −15 °C) under different humic acid (HA), zinc (Zn), and combined applications. Panels (A–H) represent individual treatment combinations as indicated above each panel. No_HA/No_Zn: no application HA and Zn; SP_HA: seed priming with HA; SP_Zn: seed priming with Zn; SA_HA: soil application of HA; SA_Zn: soil application of Zn; SA_HA+Zn: soil application of HA and Zn; SP_HA+SA_Zn: seed priming with HA and soil application of Zn; SP_Zn+SA_HA: seed priming with Zn and soil application of HA. Error bars represent the standard error of the mean (SEM). CS indicates the main effect of cold stress, Cv indicates cultivar effect, and CS × Cv indicates their interaction, based on ANOVA analysis (*P < 0.05; **P < 0.01; ns, not significant). Color coding of bars is consistent across all panels to facilitate comparison of temperature responses among treatments.

The results of the variance analysis for MDI across eight HA and Zn applications in oat varieties exposed to varying degrees of low temperature are presented in Fig. 1. In plants under low-temperature stress, differences between cultivars were statistically insignificant in the SP_Zn, SA_HA, SA_Zn, SP_HA+SA_Zn, and SP_Zn+SA_HA applications, whereas they were significant in the other applications. The present study found that low-temperature stress was significant across all applications. MDI values, which are approximately 90% under normal temperature conditions without HA and Zn (No_HA/No_Zn), decreased by almost half at 4 °C, falling to 53.17% in the cv. Albatros and 32.93% in the cv. Checota. Notably, the decline in MDI of the Albatros at temperatures below −10 °C was greater than the MDI of the Checota at temperatures above zero. At a temperature of −15 °C, the MDI value of the Checota decreased to 13% (Fig. 1A).

When HA was applied as seed priming (SP_HA), oat plants tolerated 4 °C and maintained MDI. MDI, which was over 65% at 0 °C, decreased by half in both cultivars at temperatures below zero, falling to approximately 20% at −15 °C (Fig. 1B). In Zn application as seed priming (SP_Zn), the Checota had an MDI above 86% at temperatures as low as −5 °C, while the Albatros had an MDI above 86% at temperatures as low as 0 °C. However, a notable decline in MDI was observed after temperatures dropped to −10 °C in both cultivars (Fig. 1C).

In the HA application to the soil (SA_HA), temperatures below 0 °C resulted in a significant decrease in MDI in both cultivars. At temperatures below zero, the highest MDI was measured at 52.67% in the Albatros at −5 °C, while the lowest MDI was measured at 22.71% in the Checota at −15 °C (Fig. 1D). The application of Zn to the soil (SA_Zn) resulted in MDI values ranging from 94.30% (25 °C) to 31.65% (−15 °C) for the Albatros and from 92.12% (25 °C) to 29.07% (−15 °C) for the Checota. The Checota showed lower MDI values than Albatros after −10 °C (Fig. 1E). When both HA and Zn were applied to the soil (SA_HA+Zn) in a combined manner, both cultivars exhibited an MDI value exceeding 88% at temperatures as low as −5 °C. At a temperature of −5 °C, Checota’s MDI value was 66.76%, while Albatros’ was 34.44%. However, at temperatures of −10 °C and −15 °C, Albatros showed a higher MDI value (Fig. 1F).

The application of seed priming with HA and Zn to the soil (SP_HA+SA_Zn) protected MDI down to −5 °C. However, at −10 °C, MDI sharply declined in both cultivars. At temperatures of 4 °C, 0 °C, and −5 °C, Checota’s MDI value was higher, while at −10 °C and −15 °C, Albatros’ MDI value was comparatively higher (Fig. 1G). The application of Zn *via* seed priming, combined with HA soil application (SP_Zn+SA_HA), resulted in MDI values exceeding 90% in both cultivars, even at −10 °C. At a temperature of −15 °C, Checota’s MDI value decreased to 23.88%, while Albatros’s value decreased to 4.73% (Fig. 1H).

### Relative water content (RWC) of oat plants under low temperature stress

**Figure 2:**
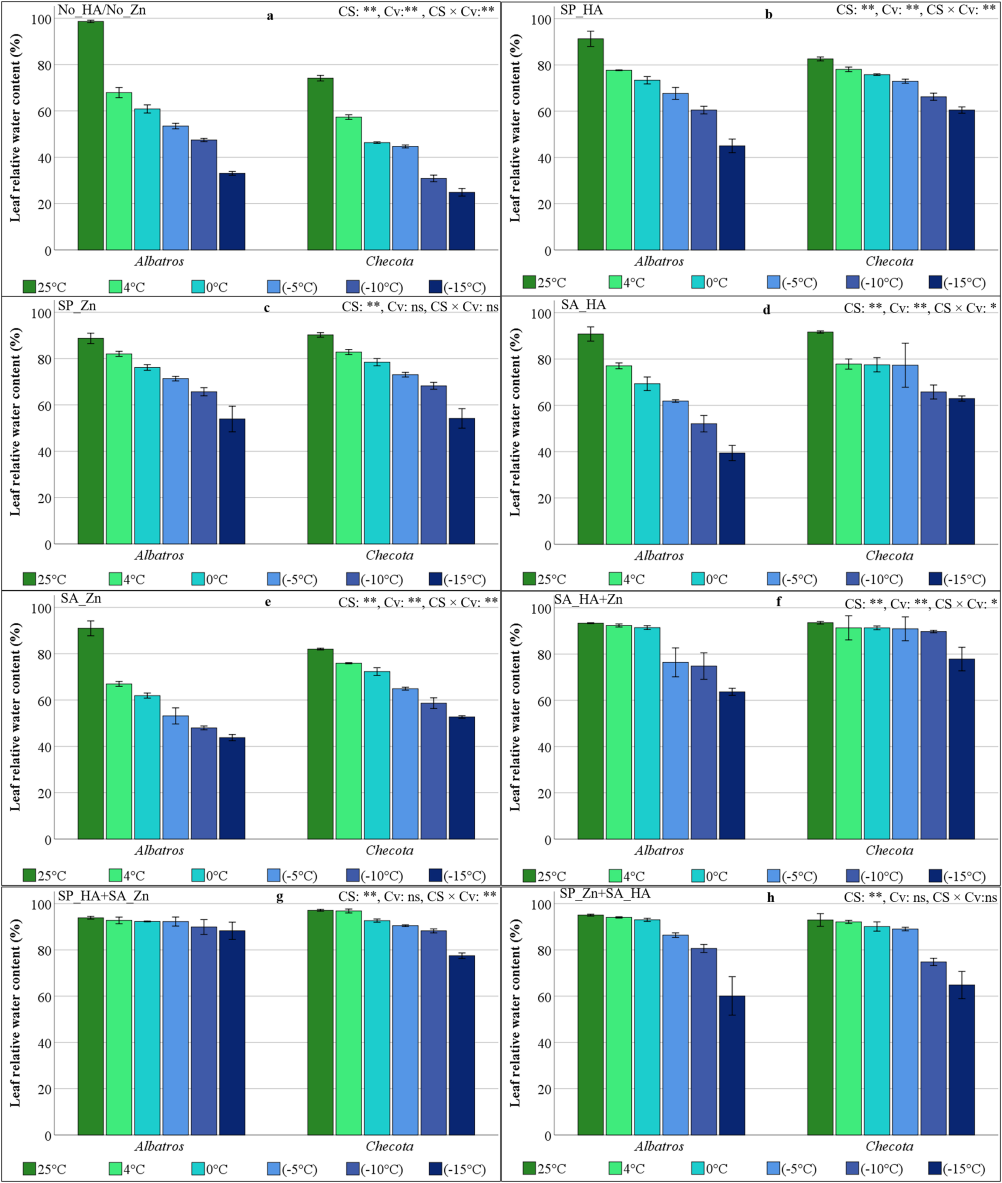
Relative water content (%) of two oat cultivars (Albatros and Checota) exposed to decreasing temperatures (25 °C, 4 °C, 0 °C, −5 °C, −10 °C and −15 °C) under different humic acid (HA), zinc (Zn), and combined applications. Panels (A–H) represent individual treatment combinations as indicated above each panel. No_HA/No_Zn: no application HA and Zn; SP_HA: seed priming with HA; SP_Zn: seed priming with Zn; SA_HA: soil application of HA; SA_Zn: soil application of Zn; SA_HA+Zn: soil application of HA and Zn; SP_HA+SA_Zn: seed priming with HA and soil application of Zn; SP_Zn+SA_HA: seed priming with Zn and soil application of HA. Error bars represent the standard error of the mean (SEM). CS indicates the main effect of cold stress, Cv indicates cultivar effect, and CS × Cv indicates their interaction, based on ANOVA analysis (*P < 0.05; **P < 0.01; ns, not significant). Color coding of bars is consistent across all panels to facilitate comparison of temperature responses among treatments.

The effect of cold stress (CS) on the RWC in oat plants was statistically significant at the 1% level across all treatments. At the same time, differences between cultivars (Cv) were insignificant in the SP_HA+SA_Zn and SP_Zn+SA_HA. The CS × Cv interaction was found to be statistically insignificant in the SP_Zn+SA_HA (Fig. 2). In normal temperature conditions during the No_HA/No_Zn, the RWC value, which was 98.64% in the Albatros and 74.14% in the Checota, decreased rapidly at low temperatures. At −15 °C, a decline was observed in the Albatros, reaching 33%, while the Checota recorded a 25% decrease (Fig. 2A).

The SP_HA resulted in a lower decrease in RWC at low temperatures. The lowest recorded RWC was 45% in the Albatros. Despite Checota having a lower RWC under normal temperature conditions, its water content was higher than that of Albatros at low temperatures (Fig. 2B). SP_Zn also appeared to be effective in maintaining water content in oat leaves. Water content, which reached 90% at normal temperatures, decreased to around 54% at −15 °C (Fig. 2C).

In the SA_HA, the RWC value of oat plants at normal temperature was around 90%. In the Checota, RWC decreased to 77% at 4 °C, 0 °C, and −5 °C. In the Albatros, RWC was at this level at 4 °C. With the SA_HA, the Checota had an RWC above 60% even at very low temperatures such as −10 °C and −15 °C. However, Albatros had an RWC that declined to 39.42% at −15 °C (Fig. 2D). As a result of SA_Zn, the RWC value of the Albatros decreased from 90.96% at normal temperature to 66.96% at 4 °C and then gradually decreased to its lowest value of 43.78% at −15 °C. In contrast, the RWC value of the Checota decreased from 82% at normal temperature conditions to 52.71% at −15 °C, with a synchronous decrease of approximately 6% on average (Fig. 2E). In the SA_HA+Zn, RWC values ranged from 93.52% (25 °C) to 77.83% (−15 °C) in the Checota, while in the Albatros, they ranged from 93.34% (25 °C) to 63.66% (−15 °C). Water loss associated with temperature decreases was lower (Fig. 2F).

SP_HA+SA_Zn resulted in the Albatros maintaining an 88.26% RWC even at −15 °C. The RWC value of the Checota, however, dropped to 77.49% at −15 °C (Fig. 2G). In the SP_Zn+SA_HA, the Albatros had an RWC of 93% or higher at 25 °C, 4 °C, and 0 °C, while the RWC values of the Checota at the same temperatures were 92.9%, 92.1%, and 90.1%, respectively. At a temperature of −10 °C, a more pronounced water loss was observed in Checota, while at −15 °C, the loss was greater in Albatros (Fig. 2H).

### Turgor loss (TL) of oat plants under low temperature stress

**Figure 3:**
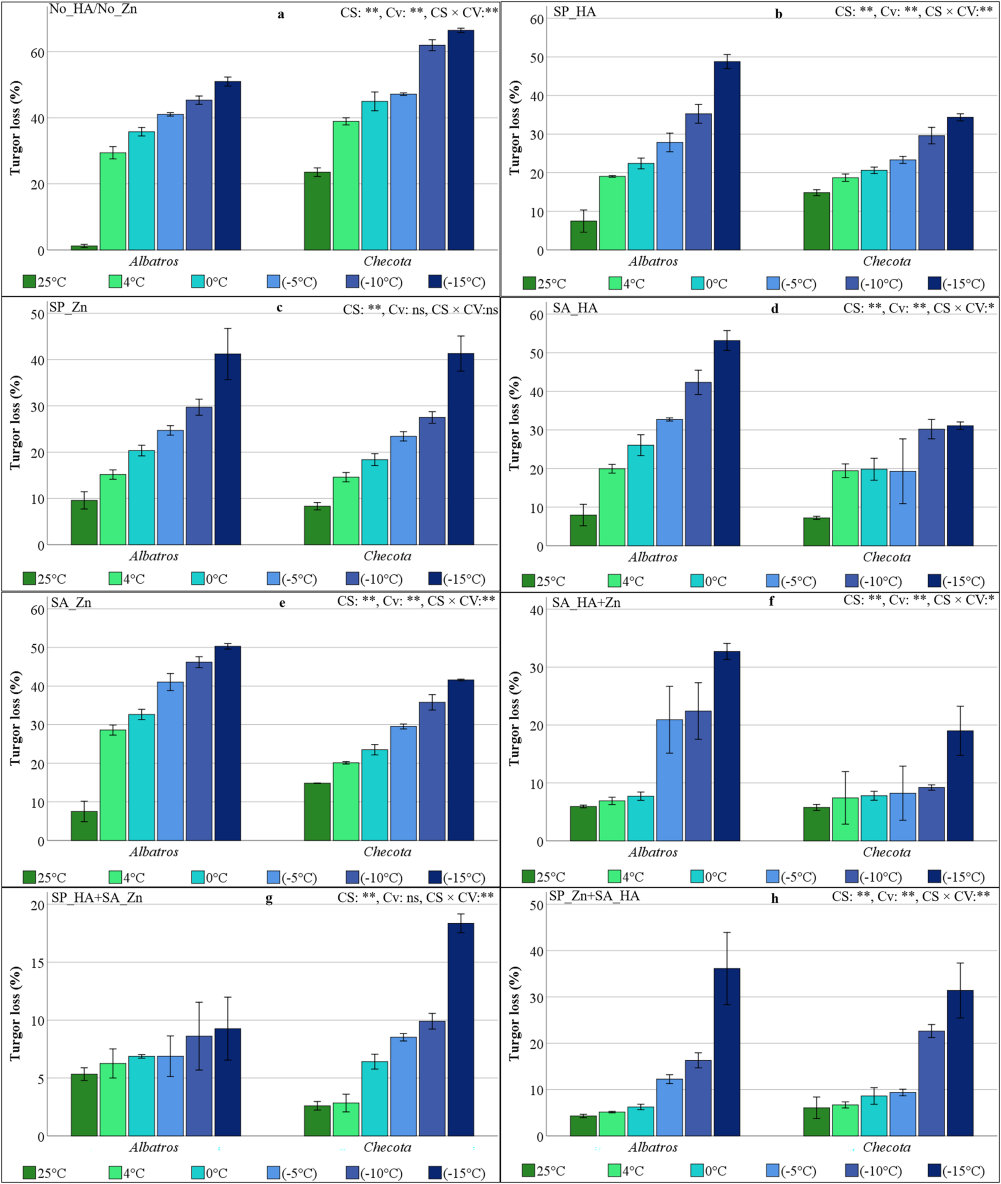
Turgor loss (%) of two oat cultivars (Albatros and Checota) exposed to decreasing temperatures (25 °C, 4 °C, 0 °C, −5 °C, −10 °C and −15 °C) under different humic acid (HA), zinc (Zn), and combined applications. Panels (A–H) represent individual treatment combinations as indicated above each panel. No_HA/No_Zn: no application HA and Zn; SP_HA: seed priming with HA; SP_Zn: seed priming with Zn; SA_HA: soil application of HA; SA_Zn: soil application of Zn; SA_HA+Zn: soil application of HA and Zn; SP_HA+SA_Zn: seed priming with HA and soil application of Zn; SP_Zn+SA_HA: seed priming with Zn and soil application of HA. Error bars represent the standard error of the mean (SEM). CS indicates the main effect of cold stress, Cv indicates cultivar effect, and CS × Cv indicates their interaction, based on ANOVA analysis (*P < 0.05; **P < 0.01; ns, not significant). Color coding of bars is consistent across all panels to facilitate comparison of temperature responses among treatments.

The significance levels obtained from the variance analysis conducted for each HA and Zn application, CS, Cv, and their interaction were equivalent to those for RWC (Fig. 3). In oat plants without HA or Zn, TL increased rapidly in parallel with the decrease in temperature, rising from 1.23% to 50.96% in the Albatros and ranging from 23.54% to 66.46% in the Checota (Fig. 3A). A parallel phenomenon to No_HA/No_Zn was observed in the SP_HA, yet TL was comparatively minimal, with the most substantial loss (48.79%) recorded in the Albatros at −15 °C. This value was followed by the Albatros exposed to −10 °C with 35.25%. The TL of the Checota at −15 °C was 34.34% (Fig. 3B). In the SP_Zn, TL showed a similar pattern in both cultivars, with values ranging from 8% to 41% and decreasing in parallel with decreasing temperature (Fig. 3C).

The Albatros treated with HA from the soil (SA_HA) had a greater TL than the Checota. The Checota exhibited TL values that were nearly identical (approximately 19%) at the initial low temperature thresholds (4 °C, 0 °C, and −5 °C). These values were also almost equivalent (approximately 30%) at −10 °C and −15 °C. However, the TL of Albatros accelerated at lower temperatures (Fig. 3D). In SA_Zn, both cultivars showed a comparable TL response to low temperatures; however, the increase in TL with decreasing temperature was more pronounced in Albatros (Fig. 3E). When both HA and Zn were applied to the soil (SA_HA+Zn), Albatros’ TL was similar at temperatures above zero but suddenly increased. The maximum TL was observed in Albatros at −15 °C, with a value of 32.7%. Checota’s TL, on the other hand, remained below 10% and similar to each other up to −15 °C. It reached 19% at −15 °C (Fig. 3F).

In SP_Zn+SA_HA, Albatros demonstrated a more stable turgor state. However, in this application, Checota rapidly lost its turgor at −15 °C, reaching a maximum of 18.36% (Fig. 3G). In SP_Zn+SA_HA, Albatros exhibited minimal TL, with a maximum decline of −5 °C. Turgor loss, which reaches 12% at −5 °C, increases to 36% at −15 °C. The turgor status of the Checota, however, was higher than that of the Albatros at −10 °C under this application but lower at −15 °C (Fig. 3H).

### Proline content of oat plants under low temperature stress

**Figure 4:**
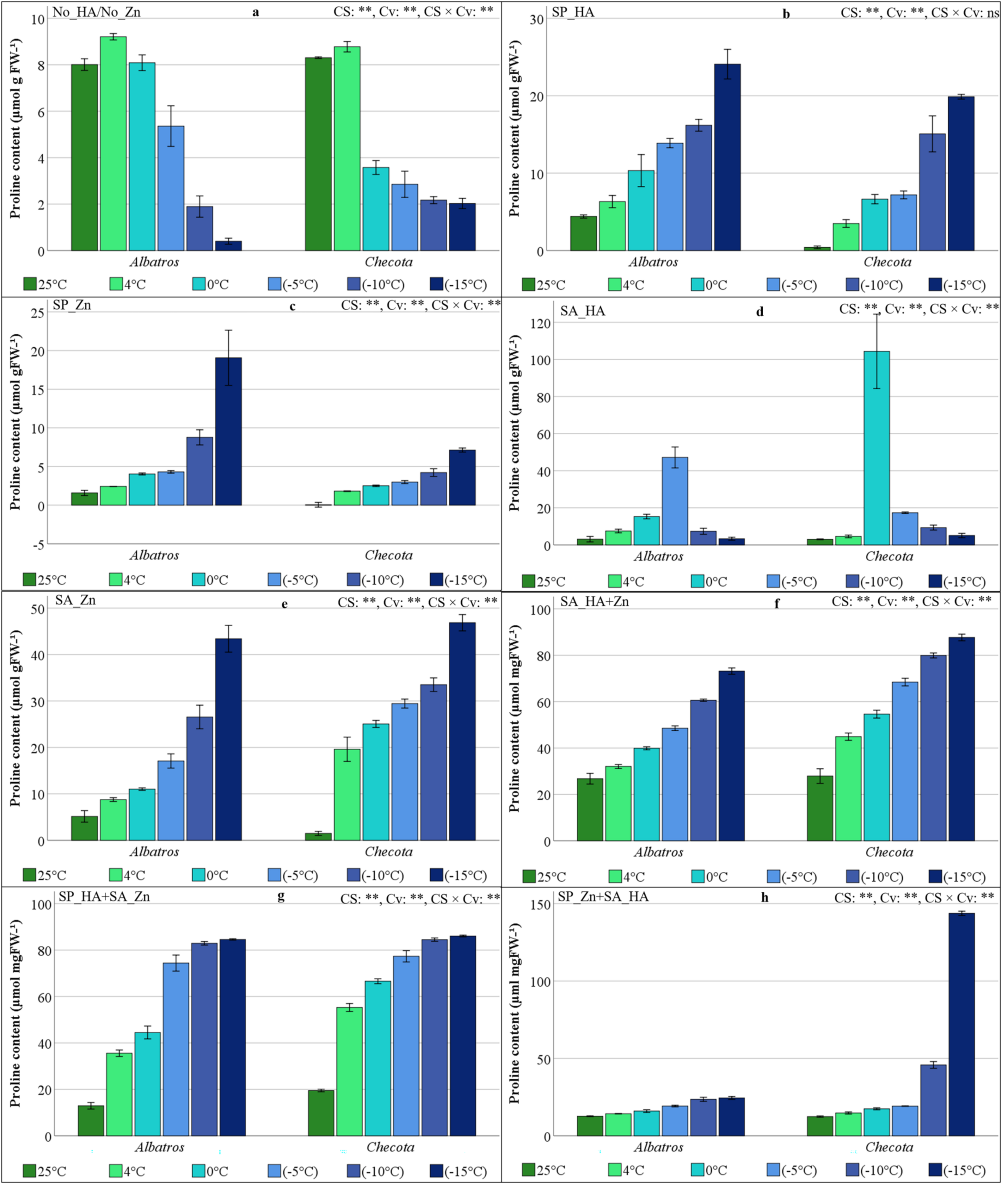
Proline content (µmol gFW −1) of two oat cultivars (Albatros and Checota) exposed to decreasing temperatures (25 °C, 4 °C, 0 °C, −5 °C, −10 °C and −15 °C) under different humic acid (HA), zinc (Zn), and combined applications. Panels (A–H) represent individual treatment combinations as indicated above each panel. No_HA/No_Zn: no application HA and Zn; SP_HA: seed priming with HA; SP_Zn: seed priming with Zn; SA_HA: soil application of HA; SA_Zn: soil application of Zn; SA_HA+Zn: soil application of HA and Zn; SP_HA+SA_Zn: seed priming with HA and soil application of Zn; SP_Zn+SA_HA: seed priming with Zn and soil application of HA. Error bars represent the standard error of the mean (SEM). CS indicates the main effect of cold stress, Cv indicates cultivar effect, and CS × Cv indicates their interaction, based on ANOVA analysis (**P < 0.01; ns, not significant). Color coding of bars is consistent across all panels to facilitate comparison of temperature responses among treatments.

The significance levels obtained from the variance analysis conducted for each HA and Zn application, CS, Cv, and their interaction were statistically significant (Fig. 4). In the absence of HA and Zn, cold stress reduced proline content. In both cultivars, the proline content increased slightly at 4 °C and gradually decreased from 0 °C onwards. While proline levels were found to be relatively high in Albatros, they were quite low at −10 °C and −15 °C (Fig. 4A). The SP_HA treatment resulted in an increase in proline content, parallel to the decrease in temperature, in both cultivars. The proline content of Albatros was consistently higher than that of Checota under all temperature conditions (Fig. 4B). The SP_Zn showed a pattern similar to that of SP_HA in terms of proline content, despite its low levels. The elevated proline content in Albatros was quite pronounced at −15 °C (Fig. 4C).

In oat cultivars cultivated with SA_HA, proline content increased up to −5 °C in Albatros, reaching its highest level (47.17 µmol g^−1^FW), and then rapidly decreased, falling back to a level close to 25 °C at −15 °C. In Checota, the proline content peaked at 0 °C (104.33 µmol g^−1^FW), decreased six-fold at −5 °C, and dropped to 5.07 µmol g^−1^FW at −15 °C (Fig. 4D). The proline content in oat cultivars cultivated with SA_Zn ranged from 5.16 µmol g^−1^FW to 43.40 µmol g^−1^FW in Albatros and from 1.50 µmol g^−1^FW to 46.85 µmol g^−1^FW in Checota. Proline content increased as the temperature decreased. In the case of Albatros, the increase was gradual. However, in Checota, when the normal temperature conditions were reduced to 4 °C, resulting in a 13-fold increase (Fig. 4E). The proline content in oat cultivars grown with SA_HA+Zn ranged from 26.77 µmol g^−1^FW (Albatros at 25 °C) to 87.67 µmol g^−1^FW (Checota at −15 °C). Proline content increased rhythmically as temperature decreased (Fig. 4F).

The proline content in oat plants treated with SP_HA+SA_Zn was relatively higher in the Checota under all temperature conditions. In both cultivars, the proline content increased approximately threefold when the temperature was lowered from 25 °C to 4 °C (Fig. 4G). The content of proline in oat plants applied with SP_Zn+SA_HA increased gradually from 12.64 µmol g^−1^FW at 25 °C to 24.46 µmol g^−1^FW at −15 °C in the Albatros. The proline content of the Checota demonstrated a close to identical response to Albatros down to −5 °C but increased rapidly to 45.78 µmol g^−1^FW at −10 °C and reached its maximum level of 143.67 µmol g^−1^FW at −15 °C (Fig. 4H). This value represents the maximum proline content observed across all applications.

### CBF1 gene expressions of oat plants under low temperature stress

**Figure 5:**
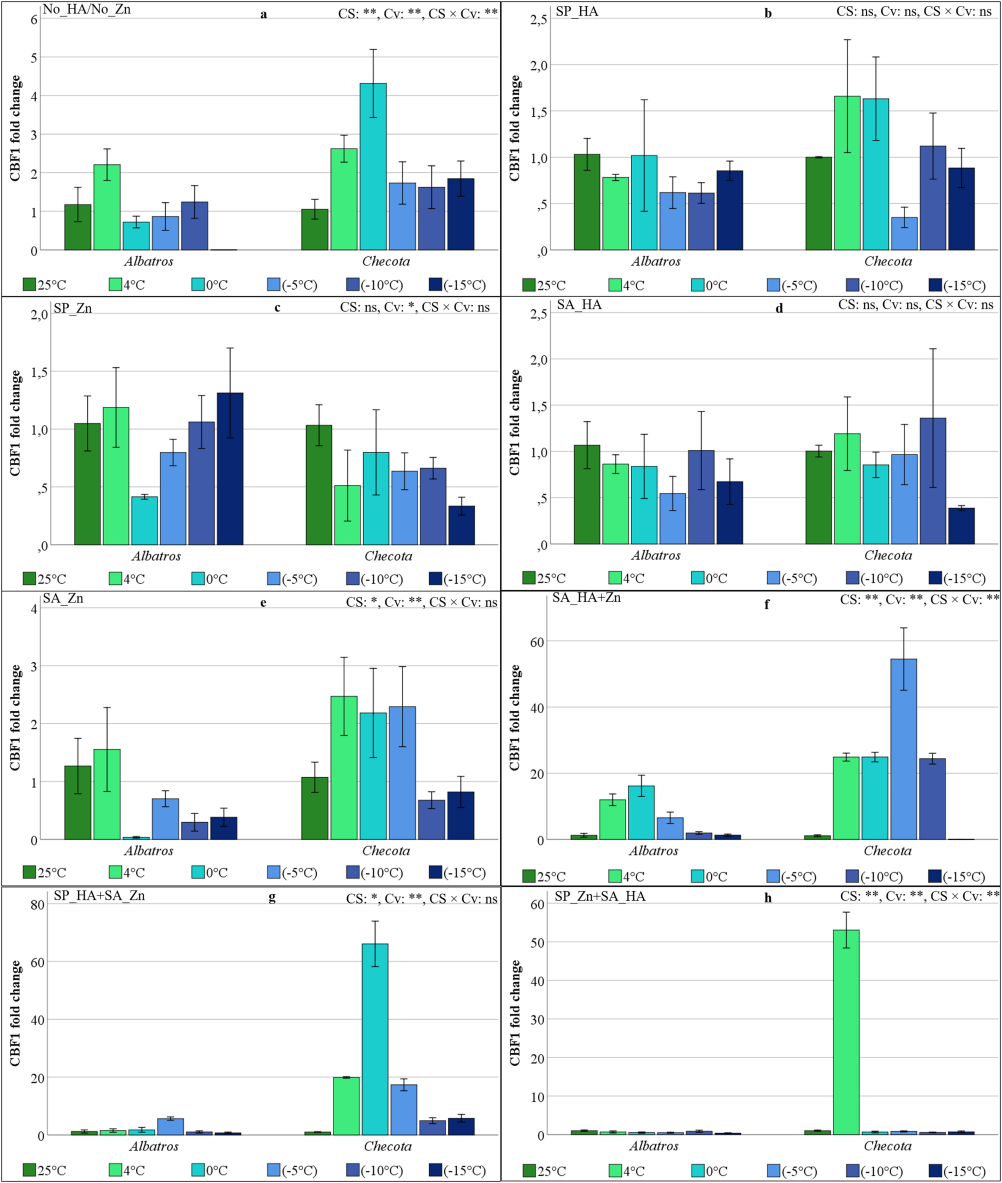
CBF1 gene expression (fold change) of two oat cultivars (Albatros and Checota) exposed to decreasing temperatures (25 °C, 4 °C, 0 °C, −5 °C, −10 °C and −15 °C) under different humic acid (HA), zinc (Zn), and combined applications. Panels (A–H) represent individual treatment combinations as indicated above each panel. No_HA/No_Zn: no application HA and Zn; SP_HA: seed priming with HA; SP_Zn: seed priming with Zn; SA_HA: soil application of HA; SA_Zn: soil application of Zn; SA_HA+Zn: soil application of HA and Zn; SP_HA+SA_Zn: seed priming with HA and soil application of Zn; SP_Zn+SA_HA: seed priming with Zn and soil application of HA. Error bars represent the standard error of the mean (SEM). CS indicates the main effect of cold stress, Cv indicates cultivar effect, and CS × Cv indicates their interaction, based on ANOVA analysis (*P < 0.05; **P < 0.01; ns, not significant). Color coding of bars is consistent across all panels to facilitate comparison of temperature responses among treatments.

The statistical analysis revealed that CS, Cv, and their interaction were significant for CBF1 gene expression in No_HA/No_Zn, SA_HA+Zn, and SP_Zn+SA_HA for CBF1 gene expression. However, these variables were found to be insignificant in the SP_HA, SP_Zn, and SA_HA (Fig. 5). In the absence of HA and Zn, the CBF1 gene expression in the Albatros increased at 4 °C, decreased at 0 °C, increased slightly at −5 °C and −10 °C, and was lost at −15 °C. In the Checota, the CBF1 gene was expressed at higher levels at 4 °C, reached its maximum level at 0 °C, and then decreased by approximately 2-fold at temperatures below 0 °C, remaining at similar levels (Fig. 5A). The SP_HA, SP_Zn, and SA_HA resulted in insignificant and fluctuating changes in CBF1 gene expression in both cultivars (Figs. 5B, 5C, and 5D). The CBF1 gene expression levels were minimal in the Albatros under the SA_Zn, SP_HA+SA_Zn, and SP_Zn+SA_HA. These levels remained consistent across all temperature conditions. The CBF1 gene expression levels in the Checota exhibited an approximately twofold increase at temperatures of 4 °C, 0 °C, and −5 °C in comparison to 25 °C, −10 °C, and −15 °C. This increase was observed in the SA_Zn, and the expression levels remained consistent at these temperatures (Fig. 5E). In the SA_HA+Zn, the Albatros resulted in exhibiting low CBF1 gene expression at 25 °C, −10 °C, and −15 °C. However, the expression levels increased approximately 9-fold at 4 °C, 12.5-fold at 0 °C, and 5-fold at −5 °C, respectively, compared to optimal temperature. Notably, the Checota exhibited a 22.6-fold increase at 4 °C and 0 °C, a 49.5-fold increase at −5 °C, and a 22.2-fold increase at −10 °C compared to normal temperatures (Fig. 5F). The SP_HA+SA_Zn increased Checotan’s CBF1 gene expression by approximately 20-fold at 4 °C, 66-fold at 0 °C, 17-fold at −5 °C, and 5-fold at −10 and −15 °C compared to normal temperature (Fig. 5G). The SP_Zn+SA_HA increased the expression of the CBF1 gene in Checota by approximately 53-fold at 4 °C compared to normal temperature. However, this effect was not observed under other temperature conditions (Fig. 5H).

### CBF2 gene expressions of oat plants under low temperature stress

**Figure 6:**
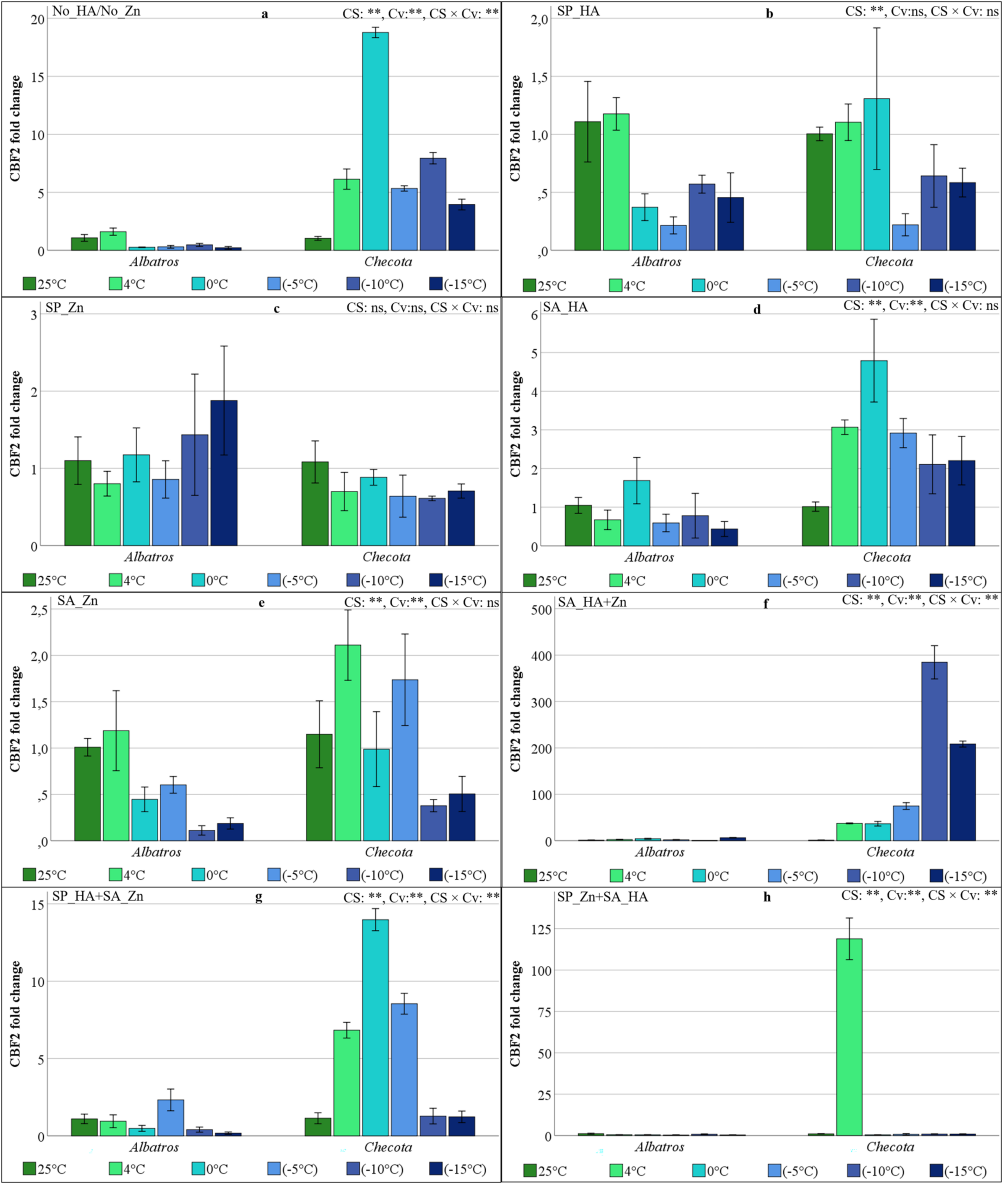
CBF2 gene expression (fold change) of two oat cultivars (Albatros and Checota) exposed to decreasing temperatures (25 °C, 4 °C, 0 °C, −5 °C, −10 °C and −15 °C) under different humic acid (HA), zinc (Zn), and combined applications. Panels (A–H) represent individual treatment combinations as indicated above each panel. No_HA/No_Zn: no application HA and Zn; SP_HA: seed priming with HA; SP_Zn: seed priming with Zn; SA_HA: soil application of HA; SA_Zn: soil application of Zn; SA_HA+Zn: soil application of HA and Zn; SP_HA+SA_Zn: seed priming with HA and soil application of Zn; SP_Zn+SA_HA: seed priming with Zn and soil application of HA. Error bars represent the standard error of the mean (SEM). CS indicates the main effect of cold stress, Cv indicates cultivar effect, and CS × Cv indicates their interaction, based on ANOVA analysis (**P < 0.01; ns, not significant). Color coding of bars is consistent across all panels to facilitate comparison of temperature responses among treatments.

The effect of cold stress on CBF2 gene expression in HA and Zn treatments was insignificant in SP_Zn. The variation in CBF2 gene expression between cultivars was not significant in SP_HA, while the CS × Cv interaction was not significant in SP_HA, SP_Zn, SA_HA, and SA_Zn (Fig. 6). The CBF2 gene expression levels were comparatively low in the Albatros, in which no treatment was applied. In the Checota, the increase in expression was 6-fold at 4 °C, 19-fold at 0 °C, 5-fold at −5 °C, 8-fold at −10 °C, and 4-fold at −15 °C compared to normal temperature (Fig. 6A). The study found that SP_Zn at 25 °C, 4 °C, and sub-zero temperatures resulted in similar CBF2 gene expression levels in both cultivars. However, Checota revealed relatively higher expression levels at 0 °C (Fig. 6B). The SP_Zn did not affect CBF2 gene expression in either cultivar and under all temperature conditions (Fig. 6C), while in SA_HA, Checota caused an increase in CBF2 gene expression at low temperatures. The highest observed fold change value was determined at 0 °C with 4.79, followed by 4 °C with 3.07 (Fig. 6D).

The pattern of change in CBF2 gene expression at low temperatures in the SA_Zn is similar in both cultivars, but Chetoca has higher fold changes (Fig. 6E). In the SA_HA+Zn, Albatros’ CBF2 gene expression ranged from 0.03 (−10 °C) to 6.34 (−15 °C), while Checota showed overexpression at temperatures below zero. The values at −10 °C and −15 °C were 384.46-fold and 208.18-fold, respectively (Fig. 6F). The SP_HA+SA_Zn resulted in the highest CBF gene expression in Albatros at −5 °C (fold change 2.3), while the fold change value around 1.2 at 25 °C, −10 °C, and −15 °C in Checota was found to be 6.8 at 4 °C, 8.5 at −5 °C, and 14 at 0 °C (Fig. 6G). The SP_Zn+SA_HA at Checota, with a fold change value of 118.9, as evidenced by an overexpression level at 4 °C (Fig. 6H).

### CBF3 gene expressions of oat plants under low temperature stress

**Figure 7:**
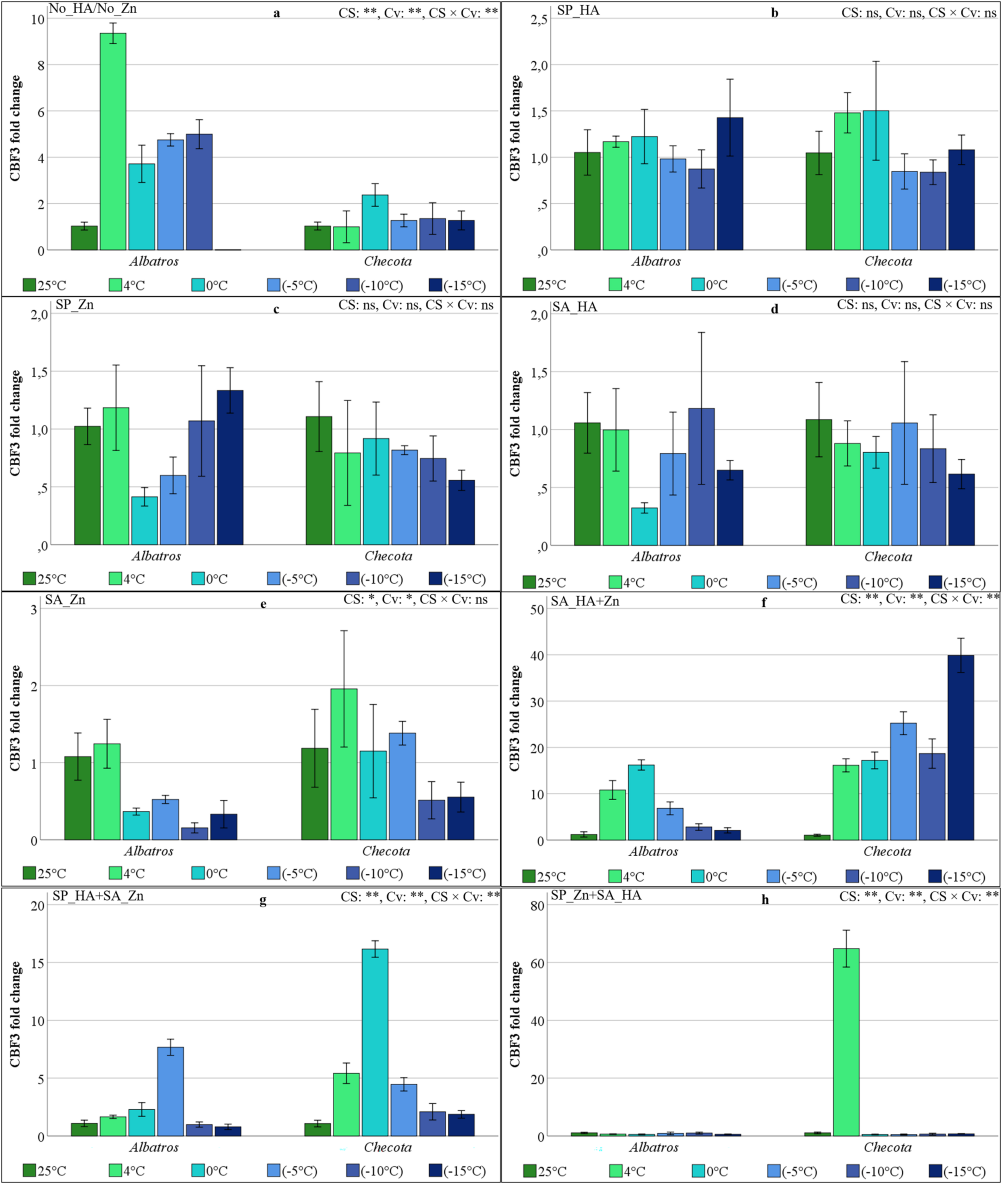
CBF3 gene expression (fold change) of two oat cultivars (Albatros and Checota) exposed to decreasing temperatures (25 °C, 4 °C, 0 °C, −5 °C, −10 °C and −15 °C) under different humic acid (HA), zinc (Zn), and combined applications. Panels (A–H) represent individual treatment combinations as indicated above each panel. No_HA/No_Zn: no application HA and Zn; SP_HA: seed priming with HA; SP_Zn: seed priming with Zn; SA_HA: soil application of HA; SA_Zn: soil application of Zn; SA_HA+Zn: soil application of HA and Zn; SP_HA+SA_Zn: seed priming with HA and soil application of Zn; SP_Zn+SA_HA: seed priming with Zn and soil application of HA. Error bars represent the standard error of the mean (SEM). CS indicates the main effect of cold stress, Cv indicates cultivar effect, and CS × Cv indicates their interaction, based on ANOVA analysis (*P < 0.05; **P < 0.01; ns, not significant). Color coding of bars is consistent across all panels to facilitate comparison of temperature responses among treatments.

Changes in CBF3 gene expression in oat plants under low temperature stress were found to be insignificant in SP_HA, SP_Zn, SA_HA, and SA_Zn (Fig. 7). In the absence of HA and Zn application, oat plants exhibited varied alterations in CBF3 gene expression among cultivars when exposed to low temperatures. The CBF3 gene expression of Albatros increased 9.3-fold at 4 °C, then decreased to 3.7-fold at 0 °C, increased to 4.8-fold at −5 °C and −10 °C, and disappeared completely at −15 °C (Fig. 7A). The expression levels of the CBF3 gene in the SP_HA, SP_Zn, SA_HA, and SA_Zn were found to be low in both cultivars and exhibited an up-and-down expression pattern (Figs. 7B–7E). In the SA_HA+Zn, the CBF3 gene expression of the Albatros ranged from 1.22 (25 °C) to 16.21 (0 °C). The expression level increased with chilling temperature and gradually decreased as the temperature fell below zero. The CBF3 gene expression in Checota was found to be higher. A decline in temperature to 4 °C increased expression level by 15.5-fold compared to normal temperature conditions, reaching a fold change value of 25.23 at −5 °C. Despite a subsequent decline at −10 °C, the maximum recorded value was 39.87 at −15 °C (Fig. 7F).

In the SP_HA+SA_Zn, the maximum CBF3 fold change value in Albatros was recorded at −5 °C, with a value of 7.67. The CBF3 gene expression value, which was 2.29 at 0 °C, was low and similar at the other temperatures. The lowest CBF3 gene expression levels in Checota were determined at 25 °C, −15 °C, and −10 °C. The maximum value was observed at 0 °C as 16.16, with approximately 5-fold changes at 4 °C and −5 °C (Fig. 7G). In the experiment involving SP_Zn+SA_HA, no temperature-dependent change was observed in CBF3 gene expression levels in either cultivar. However, Checota showed overexpression of the CBF3 gene at 4 °C, with a value of 64.79 (Fig. 7H).

### CBF4 gene expressions of oat plants under low temperature stress

**Figure 8:**
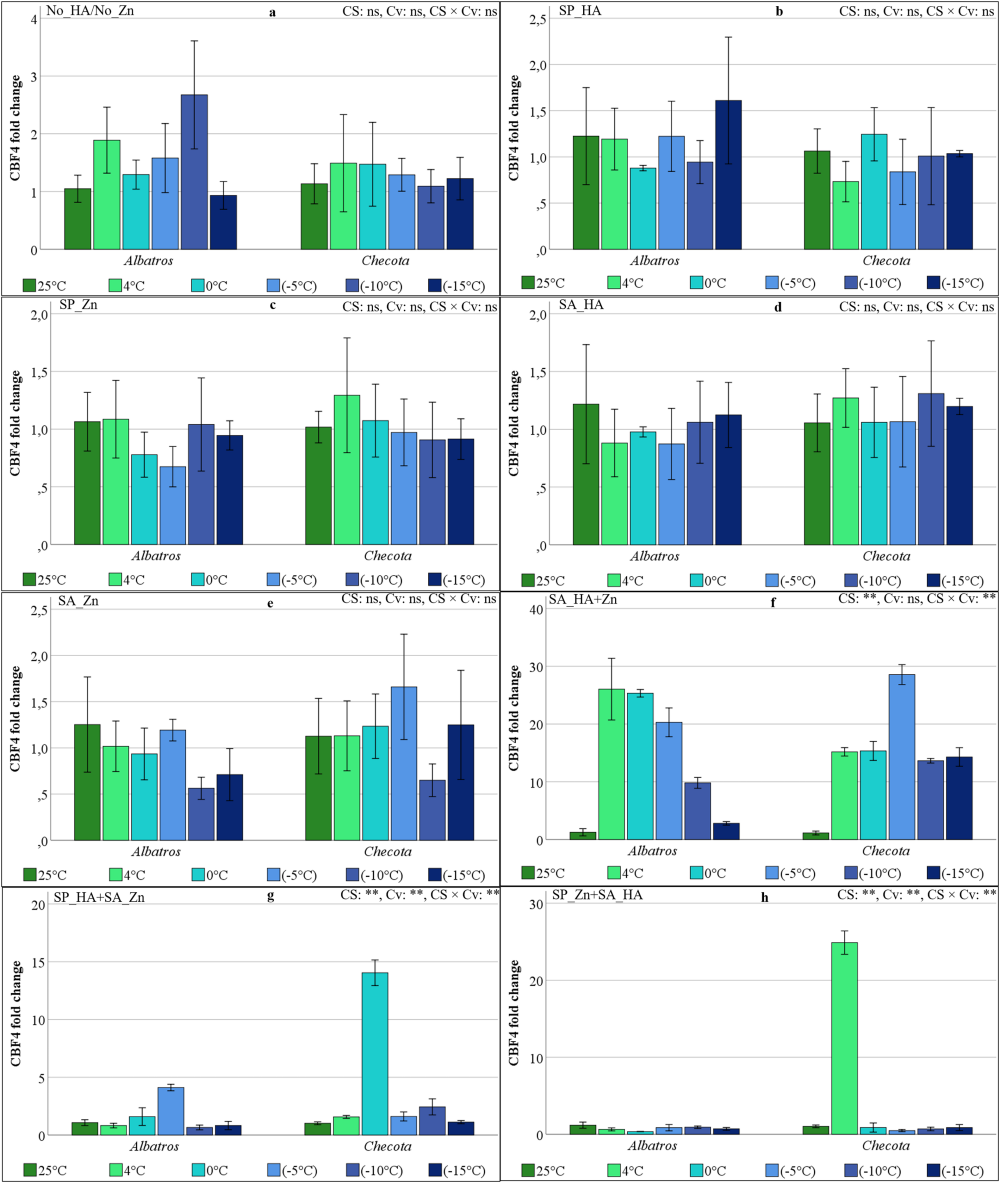
CBF4 gene expression (fold change) of two oat cultivars (Albatros and Checota) exposed to decreasing temperatures (25 °C, 4 °C, 0 °C, −5 °C, −10 °C and −15 °C) under different humic acid (HA), zinc (Zn), and combined applications. Panels (A–H) represent individual treatment combinations as indicated above each panel. No_HA/No_Zn: no application HA and Zn; SP_HA: seed priming with HA; SP_Zn: seed priming with Zn; SA_HA: soil application of HA; SA_Zn: soil application of Zn; SA_HA+Zn: soil application of HA and Zn; SP_HA+SA_Zn: seed priming with HA and soil application of Zn; SP_Zn+SA_HA: seed priming with Zn and soil application of HA. Error bars represent the standard error of the mean (SEM). CS indicates the main effect of cold stress, Cv indicates cultivar effect, and CS × Cv indicates their interaction, based on ANOVA analysis (**P < 0.01; ns, not significant). Color coding of bars is consistent across all panels to facilitate comparison of temperature responses among treatments.

The alterations in CBF4 gene expression in oat plants in response to low-temperature stress were not statistically significant in the No_HA/No_Zn, SP_HA, SP_Zn, SA_HA, and SA_Zn, with an approximate average change of 0.6–2.6 (Figs. 8A–8E). In the SA_HA+Zn, the CBF4 gene expression levels of Albartos and Checota were similar. The statistical analysis revealed that the CS and CS × Cv interaction was statistically significant at the 1% level. The two cultivars showed the lowest CBF4 gene expression at 25 °C, whereas Albatros showed the highest expression at 4 °C, with a fold change of 26.04. Thereafter, CBF4 expression gradually decreased. In the Checota, the highest expression level was 28.55 at −5 °C, and it was close to the other temperature levels, with approximately 15-fold changes (Fig. 8F). In the SP_HA+SA_Zn, Albartos attained the maximum CBF4 gene expression value (4.11) at −5 °C. Conversely, Checota showed the highest CBF4 gene expression at 0 °C (14.04), followed by 2.43 at −10 °C. At varying temperatures, both cultivars exhibited low CBF4 gene expression (Fig. 8G). In the SP_Zn+SA_HA, the CBF4 gene expression level reached its maximum at 24.88 in Checota at 4 °C. The statistical analysis revealed that CS, Cv, and their interaction were all statistically significant. At varying temperatures, the CBF4 gene expression levels in both cultivars ranged from 0 to 1, with a similar pattern (Fig. 8H).

### VRN1 gene expressions of oat plants under low temperature stress

**Figure 9:**
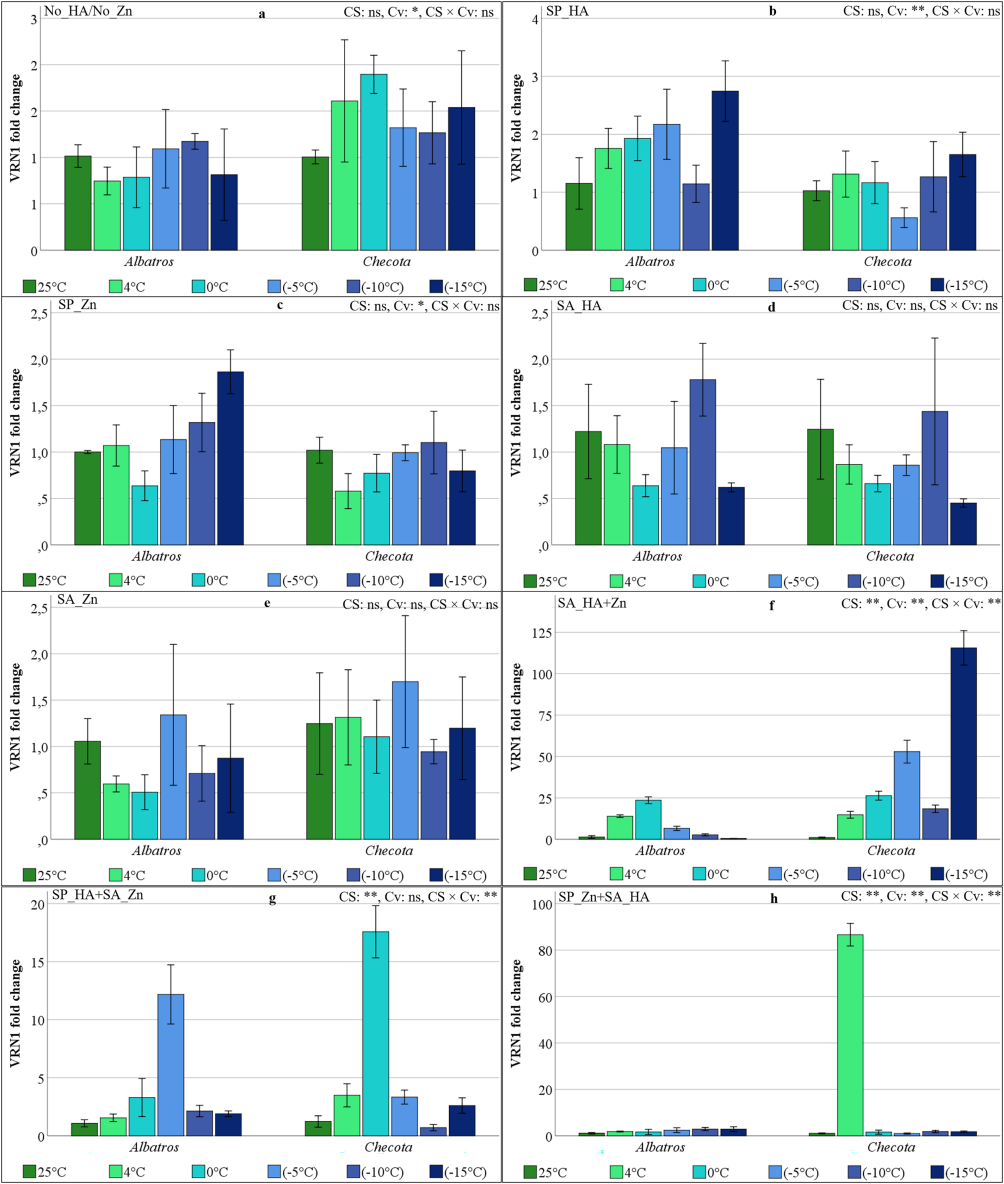
VRN1 gene expression (fold change) of two oat cultivars (Albatros and Checota) exposed to decreasing temperatures (25 °C, 4 °C, 0 °C, −5 °C, −10 °C and −15 °C) under different humic acid (HA), zinc (Zn), and combined applications. Panels (A–H) represent individual treatment combinations as indicated above each panel. No_HA/No_Zn: no application HA and Zn; SP_HA: seed priming with HA; SP_Zn: seed priming with Zn; SA_HA: soil application of HA; SA_Zn: soil application of Zn; SA_HA+Zn: soil application of HA and Zn; SP_HA+SA_Zn: seed priming with HA and soil application of Zn; SP_Zn+SA_HA: seed priming with Zn and soil application of HA. Error bars represent the standard error of the mean (SEM). CS indicates the main effect of cold stress, Cv indicates cultivar effect, and CS × Cv indicates their interaction, based on ANOVA analysis (*P < 0.05; **P < 0.01; ns, not significant). Color coding of bars is consistent across all panels to facilitate comparison of temperature responses among treatments.

The variation in VRN1 gene expression in response to CS was not statistically significant and was minimal in all experimental groups, including No_HA/No_Zn, SP_HA, SP_Zn, SA_HA, and SA_Zn (Figs. 9A–9E). In the SA_HA+Zn experiment, both the Albatros and Checota exhibited a comparable pattern and approximate values at temperatures above zero. At below zero temperatures, VRN1 gene expression in the Albatros gradually decreased, reaching 52.9 in the Checota at −5 °C. It then exhibited a decrease to 18.4 at −10 °C and finally attained its peak value of 115.5 at −15 °C (Fig. 9F). In the SP_HA+SA_Zn, VRN1 gene expression values ranged from 0.71 (Checota at −10 °C) to 17.58 (Checota at 0 °C). In Albatros, the VRN1 fold change value, which increased as the temperature decreased and was 12.18 at −5 °C, decreased approximately six-fold at −10 °C and −15 °C (Fig. 8G). In the SP_Zn+SA_HA, VRN1 gene expression varied between 1.0 (Checota at 0 °C) and 2.9 (Albatros at −10 °C), showing an overexpression value of 86.6 at 4 °C in Checota compared to other temperatures (Fig. 9H).

### ZAT12 gene expressions of oat plants under low temperature stress

**Figure 10:**
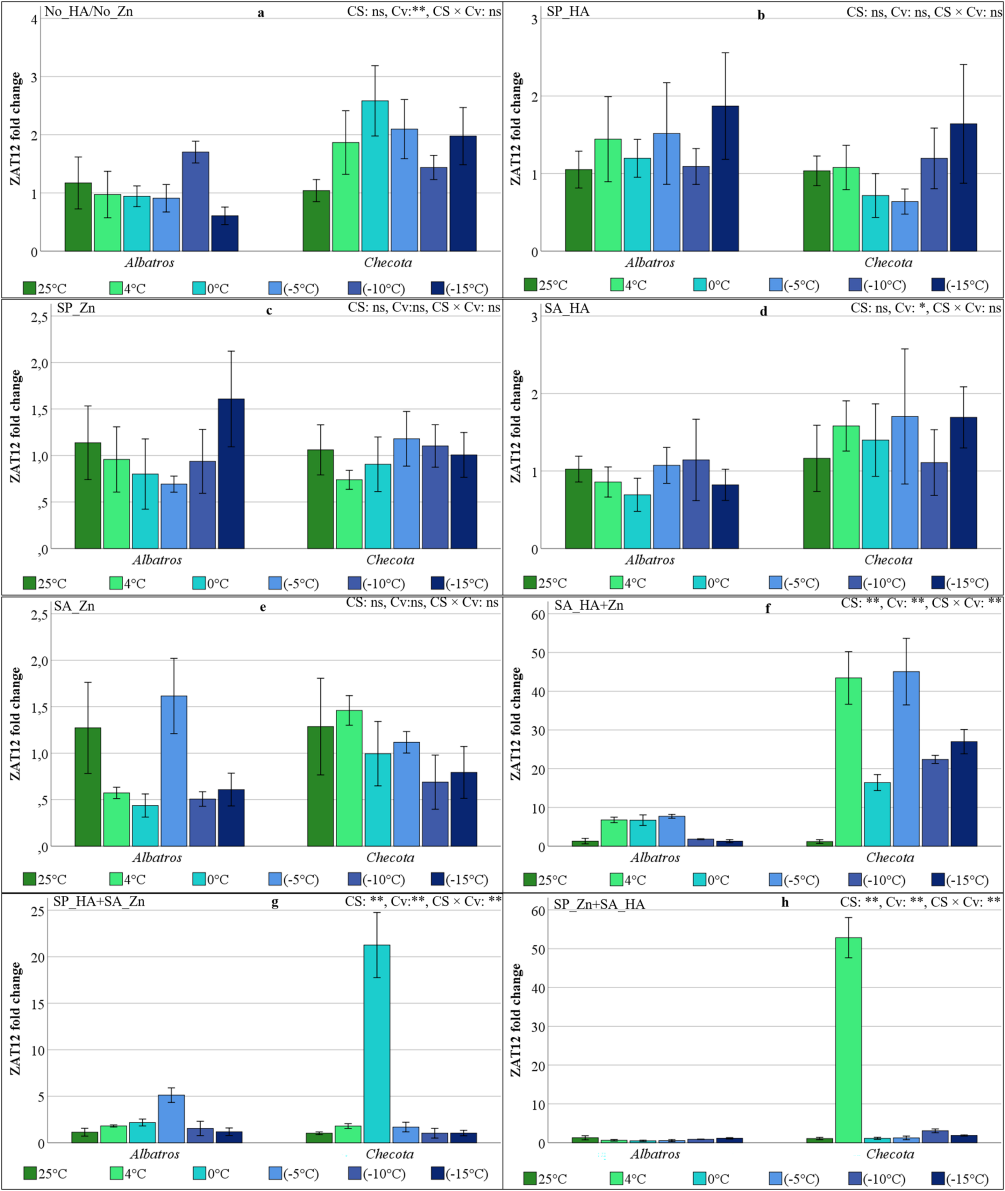
ZAT12 gene expression (fold change) of two oat cultivars (Albatros and Checota) exposed to decreasing temperatures (25 °C, 4 °C, 0 °C, −5, −10 °C and −15 °C) under different humic acid (HA), zinc (Zn), and combined applications. Panels (A–H) represent individual treatment combinations as indicated above each panel. No_HA/No_Zn: no application HA and Zn; SP_HA: seed priming with HA; SP_Zn: seed priming with Zn; SA_HA: soil application of HA; SA_Zn: soil application of Zn; SA_HA+Zn: soil application of HA and Zn; SP_HA+SA_Zn: seed priming with HA and soil application of Zn; SP_Zn+SA_HA: seed priming with Zn and soil application of HA. Error bars represent the standard error of the mean (SEM). CS indicates the main effect of cold stress, Cv indicates cultivar effect, and CS × Cv indicates their interaction, based on ANOVA analysis (*P < 0.05; **P < 0.01; ns, not significant). Color coding of bars is consistent across all panels to facilitate comparison of temperature responses among treatments.

In all experimental groups, including No_HA/No_Zn, SP_HA, SP_Zn, SA_HA, and SA_Zn, the variation in ZAT12 gene expression in response to CS was negligible and not statistically significant (Figs. 10A–10E). In the SA_HA+Zn, Albatros’ ZAT12 gene expression increased approximately 6-fold at 4 °C, 0 °C, and −5 °C compared to normal temperature and decreased again 6-fold at −10 °C and −15 °C. The ZAT12 gene expression of the Checota increased 43-fold at 4 °C, decreased 2.6-fold to 16.4 at 0 °C, increased again by 2.7-fold at −5 °C, decreased to 22.4 at −10 °C, and then increased to a 27-fold change value at −15 °C, exhibiting an up-and-down pattern (Fig. 10F). In the SP_HA+SA_Zn, Albatros’ ZAT12 gene expression gradually increased up to −5 °C and then decreased by approximately 3.4-fold. Checota’s ZAT12 gene expression levels peaked at 0 °C, with a 21.3-fold change value, and then rapidly decreased (Fig. 10G). In the SP_Zn+SA_HA, the 52.8-fold change in ZAT12 gene expression observed at 4 °C in Checota was higher than that of the others. This value was subsequently followed by Checota’s gene expression at −10 °C, which is 3.1 (Fig. 10H).

## Discussion

### Membrane stability and leaf water status of oat plants under low temperature stress

Recent progress in our understanding of the physiological and molecular mechanisms that enable plants to sense, respond to, and survive low temperatures has provided new insights into both plant adaptations and regulatory networks (Sardoei & Fazeli-Nasab, 2025). The primary areas of this research included the roles of membrane stability, leaf water status, and osmolyte accumulation in cold adaptation, as well as the effects of HA and Zn applications on these processes. At the molecular level, the focus was on CBF transcription factors that regulate gene expression in response to low temperatures, related signaling pathways, and the integration of their regulation by HA and Zn applications. At low temperatures, particularly below −5 °C, the MDI decreased significantly in all treatments and in both cultivars compared to the control (Fig. 1). The MDI of the Checota exceeded that of the Albatros. A review of the MDI revealed that the Checota, being a winter type, sustained less damage at low temperatures and responded better to the applied treatments than the Albatros. The membrane stability of plants treated with HA or Zn to seed priming and soil application together (SP_HA+SA_Zn and SP_Zn+SA_HA) was higher than that of other treatments. In addition, Zn had been observed to be more effective than HA in preserving membrane stability. As reported by Karimi, Amini & Ghabooli (2022), Zn fertilization has been shown to help maintain membrane stability in plants under low temperature stress. Additionally, Zn has been documented to stabilize and protect the biomembrane from oxidative and peroxidative damage, loss of plasma membrane integrity, and alterations in membrane permeability (Li et al., 2013). The HA can stabilize plant cell membranes, thereby reducing electrolyte leakage and maintaining cellular homeostasis under stress (Nabi et al., 2025).

The presence of HA has been shown to play a crucial role in maintaining cellular turgor and osmotic balance. Furthermore, it has been observed that HA promotes the synthesis of suitable solutes, including proline and carbohydrates. These solutes have been demonstrated to stabilize membranes and proteins under stress conditions (Canellas et al., 2024). The decreases in leaf RWC and increases in TL associated with the decreasing temperature were lower in plants treated with HA and Zn (Figs. 2 and 3). The application of HA and Zn was found to contribute to maintaining water content and turgor in the plants. In contrast to the application of HA or Zn alone, the combined application of HA and Zn prevented the water and turgor loss associated with cold stress. The Zn application has been shown to enhance a variety of physiological processes, including chlorophyll content, stomatal conductance, RWC, and osmolyte accumulation. These processes collectively enhance growth and yield and protect leaf tissues from the deleterious effects of moisture deficiency (Kumari et al., 2022).

### Proline accumulation and its osmoprotective mechanism

A notable plant response to low temperatures is the accumulation of proline. Proline is a water-soluble amino acid that generally accumulates under stress conditions and serves as an indicator of plant resistance. Elevated levels of proline, a potent osmoprotectant, are typically associated with enhanced resistance to abiotic stress conditions (Ghosh et al., 2022). In the study, following low temperature stress treatment in oat cultivars, proline levels in the No_HA/No_Zn experiment were significantly lower than those in various HA and Zn treatments and decreased with stress (Fig. 4A). Seed treatments tended to elevate proline levels, albeit modestly, as temperatures declined. The efficacy of SP_HA was found to exceed that of SP_Zn in enhancing proline accumulation (Figs. 4B and 4C). A rise in proline content was observed at temperatures as low as −5 °C in Albatros and 0 °C in Checota following HA application to soil. However, a rapid decline in proline content was recorded at temperatures below these levels (Fig. 4D). In the SA_Zn, proline levels in both cultivars increased in parallel with the decreasing temperature and reached similar levels (Fig. 4E). In the SA_HA+Zn and SP_HA+SA_Zn treatments, proline accumulation was found to be significantly higher than other treatments, and this accumulation increased with decreasing temperature (Figs. 4F and 4G). In the SP_Zn+SA_HA, excessive proline accumulation was observed in the Checota at −15 °C (Fig. 4H). Overproduction of proline in plant cells contributes to maintaining cellular homeostasis, water uptake, osmotic adjustment, and redox balance. This, in turn, facilitates the restoration of cell structures and the reduction of oxidative damage (Ghosh et al., 2022). Li et al. (2023) reported that the application of HA in zucchini plants and Zhu et al. (2024) in melon plants protected against the adverse effects of cold stress by increasing proline accumulation. Mushtaq et al. (2023) reported that Zn protects plants from the adverse effects of stress by improving proline biosynthesis. Specifically, Zn and HA likely act as co-activators in the proline biosynthetic pathway. Zn serves as a structural component for transcription factors that upregulate Δ^−1^-pyrroline-5-carboxylate synthetase (P5CS) (Zarea & Karimi, 2023), while HA may function as a signaling molecule to suppress proline dehydrogenase (PDH) activity, thereby ensuring high intracellular proline levels for membrane stabilization and ROS scavenging (Souza et al., 2022).

### Transcriptional regulation of cold-responsive genes (CBFs, VRN1, and ZAT12)

In the present study, the expression levels of CBFs, VRN1, and ZAT12 genes were found to vary in response to low temperature stress, HA, and Zn applications, as revealed by qRT-PCR. A comprehensive examination of all gene expression levels in Albatros revealed a general pattern of lower expression compared to Checota, irrespective of the specific treatment conditions. The expression of the CBF2 gene, in particular, remained relatively stable across all treatments and at low temperatures (Fig. 6). The ICE-CBF-COR signaling pathway in plants plays a pivotal role in regulating cold stress acclimation. Cold stress is sensed by receptor proteins, which trigger a signaling cascade. This, in turn, activates the expression of CBF genes, which are then regulated. The result of this process is the up-regulation of CBF gene transcription and expression. The CBF protein binds to a cis-regulatory element (CRT/DRE) of the COR gene promoter and activates their transcription (Ritonga et al., 2021). Transcriptional regulation and post-translational modifications regulate and modify these entities at different levels of the signaling cascade, altering their expression or activity. These activities have been shown to enhance cold stress tolerance (Hwarari et al., 2022). Consequently, the enhanced expression of the genes under scrutiny in this study signifies the plant’s capacity to adapt to low temperatures and cope with stress. In the absence of HA and/or Zn application, no substantial increase in gene expression was detected at lower temperatures in the Albatros, except for the CBF3 gene. However, in the SA_HA+Zn, SP_HA+SA_Zn, and SP_Zn+SA_HA, gene expression increased in parallel with the decrease in temperature, varying by gene.

In Checota, all genes demonstrated significantly elevated relative expression levels in response to temperature reductions following soil application of HA+Zn. The SA_HA+Zn treatment in Albatros led to substantial increases in gene expression, although these increases were less pronounced than those observed in Checota. Despite the presence of analogous sequence structures and binding properties, CBFs (CBF1, CBF2, and CBF3) have distinct roles under cold stress, reflecting differences in their individual protein sequences (Guo et al., 2019). The CBF2 gene had a significantly different expression pattern. In the spring cultivar Albatros, which is sensitive to low temperatures, neither application nor temperature gradient altered gene expression. In the winter cultivar Checota, which is tolerant to low temperatures, an approximately 385-fold increase was observed at −10 °C following the SA_HA+Zn. In SP_Zn+SA_HA, the expression of the CBF2 gene in the Checota increased 119-fold at 4 °C and subsequently disappeared again at lower temperatures (Fig. 6). Consistent findings concerning CBF2 gene expression have been documented by Bräutigam et al. (2005), where AsCBF2 expression was described as highly variable and difficult to reproduce, possibly reflecting its role as a rapidly inducible regulator during specific phases of cold acclimation. Therefore, the pronounced CBF2 induction observed in Checota under SA_HA+Zn likely reflects a treatment-specific and cultivar-dependent regulatory response. All CBF genes appeared to be active in all treatments at different stages of the cold acclimation process. The expression levels of these genes generally ranged from early induction at 4 °C to predominant expression at 0 °C, −5 °C, and −10 °C. Novillo, Medina & Salinas (2007) reported that CBF2 fine-tunes the coordinated additive interaction between CBF1 and CBF3, thereby balancing the development of freezing tolerance with overall plant growth and development following cold acclimation. In contrast, the early-responding transcription factor CBF4, together with CBF2, has been reported to mediate a more precisely tuned and attenuated response to previously encountered stressors (Vyse et al., 2022). While the intricate regulatory networks of CBF transcription factors remain to be fully elucidated, their induction is known to be trigger-specific. In the present study, it was assumed that the application of HA and Zn *via* distinct methods serves as a specific CBF trigger, enhancing low-temperature tolerance in oat plants.

VRN1 was the first vernalization gene to be discovered and cloned. Its expression is increased in the stem tip meristem and leaves of winter wheat after low temperature treatment, playing a positive regulatory role in wheat heading and directing the plant from the vegetative to the reproductive phase (Yan et al., 2003; Trevaskis et al., 2007). It has also been demonstrated to play a role in cold acclimation and frost tolerance (Dhillon et al., 2010). Its role in conferring cold tolerance has been reported to involve direct binding to and modulation of the expression of CBF/DREB and other freezing tolerance pathway genes. However, the mechanisms of interaction remain unclear (Caccialupi et al., 2023). The changes in VRN1 gene expression in oat plants exposed to cold stress were similar to those observed in the CBF genes. However, the Albatros showed a marked increase in VRN1 gene expression, particularly at 0 °C and −5 °C, when HA and Zn were applied together. In the Checota, the peaks occurred at −15 °C in the SA_HA+Zn, at 0 °C in the SP_HA+SA_Zn, and at 4 °C in the SP_Zn+SA_HA (Fig. 9).

The expression of the ZAT12 gene also increased in both cultivars in experiments where HA and Zn were applied together. In the Albatros, the temperature gradually increased up to −5 °C and then decreased in the SA_HA+Zn and SP_HA+SA_Zn. In Checota, the expression profile was most pronounced in SA_HA+Zn at 4 °C and −5 °C, and although less pronounced at 0° C, −10 °C, and −15 °C, it had a higher expression level compared to normal temperatures. SP_HA+SA_Zn demonstrated significantly higher expression levels at 0 °C compared to other temperatures, and SP_Zn+SA_HA exhibited approximately 50-fold higher expression levels at 4 °C compared to other temperatures. Cold-induced regulation of the CBF regulon also involves co-regulation by the zinc finger transcription protein ZAT12. Increased expression of the ZAT12 gene has been demonstrated to alleviate low temperature stress through a convergent effect on osmotic adjustments and increased osmolyte content (Kaur et al., 2023). The complex regulation of CBF genes differs from what has been previously described, as it varies across treatments. This finding indicated that CBF factors play a multifaceted and varied role in the processes of cold acclimation and maintenance in oats, and that these factors are influenced by HA and Zn treatments.

### Synergistic effects of HA and Zn application methods on stress tolerance

The results of this study clearly demonstrated that separate application of HA and Zn, either alone or to the seed or soil, is insufficient to trigger a significant transcriptional response in AsCBFs, AsVRN1, or AsZAT12. This lack of response in the single-application groups highlights a necessary synergistic requirement between the organic signaling of HA and the constitutive/catalytic role of Zn to overcome the cold-induced gene activation threshold. The most striking finding is how different combined application methods shift the peak expression of these genes towards specific thermal ranges. The maximum expression of AsCBFs at −10 °C and AsVRN1 at −15 °C in the SA_HA+Zn group demonstrated that the soil-based combination provides sustained and robust protection. Soil-applied HA likely enhances the long-term bioavailability of Zn (Morais et al., 2022) and induces sustained systemic resistance even when temperatures drop to lethal levels. The delayed but strong induction of AsVRN1 at −15 °C demonstrates that SA_HA+Zn application strengthens the plant’s last line of defense against frost (Bräutigam, 2009). The temperature-specific peaks of AsZAT12 (highest values at 4 °C and −5 °C) further support its role as a thermal guard. In the SA_HA+Zn group, the preceding of extreme cold peaks of ZAT12 expression with CBFs and VRN1 confirmed that HA and Zn facilitate a hierarchical response: first activating ZAT12 to manage initial stress and oxidative signaling, then significantly inducing CBF regulation and VRN1 (Kaur et al., 2023) as stress escalates to −10 °C and −15 °C. These findings demonstrated that the application method of HA and Zn determines the plant’s transcription strategy.

On the contrary, when the HA and Zn were applied both seed and soil, the peak expression shifts towards higher temperatures. In the SP_HA + SA_Zn application, the highest gene expression for all genes was observed at 0 °C. This suggests that HA priming in the seed can prepare the metabolic memory of the embryo (Jatana et al., 2024), and that this, when supported by soil Zn, extends the plant’s optimal regulatory window to, but not necessarily beyond, the freezing point (0 °C). The SP_Zn+SA_HA application triggered maximum expression for all genes at 4 °C and showed minimal activity at lower temperatures. This suggests a rapid response mechanism where primed Zn in the seed provides immediate availability for zinc finger proteins such as ZAT12 (Evens, 2017), but the protection is transient and specialized for the initial onset of cold. While seed pretreatment prepares the plant for sudden and moderate cold shocks (4 °C to 0 °C), the combined application of HA and Zn to the soil is essential for withstanding extreme sub-zero conditions (−10 °C to −15 °C) due to a more stable and long-lasting nutrient-signal interaction in the rhizophospheric environment.

The transcriptional activation of AsCBFs, AsVRN1, and AsZAT12, achieved through the synergistic application of HA and Zn, was fundamentally reflected in the physiological resilience of oat plants. The stability of the ICE1-CBF-COR pathway provides a molecular scaffold that minimizes cellular damage, as evidenced by significant improvements in MDI, RWC, proline accumulation, and TL mitigation (Zhou et al., 2025). The superior performance of SA_HA+Zn and SP_HA+SA_Zn applications at extreme temperatures (−10 °C and −15 °C) confirmed the role of these biostimulants as systemic pretreatments. While a sharp decrease in RWC (falling below 40%) and a large increase in TL (exceeding 60% in some varieties) were observed in control plants (untreated with HA/Zn), the combined application of HA and Zn maintained RWC above 80% even at −15 °C. This water-retention capacity is directly linked to the extensive induction of proline, a key cryoprotectant regulated by the CBF pathway, and had peaked in these specific treatment groups. Proline accumulation acts as an osmotic buffer, effectively lowering the freezing point of the cytoplasm and reducing physical pressure on the cell walls, significantly minimizing total cell loss (Raza et al., 2023).

Furthermore, MDI data provide a clear indication of membrane protection. In control groups, cold stress led to a sharp decrease in membrane stability, while plants treated with the combination of HA and Zn maintained high stability indices even under extreme sub-zero conditions. The preservation of membrane integrity likely stems from Zn’s structural role in stabilizing biological membranes (Cakmak, 2000). The peak expression of the zinc finger protein AsZAT12 at 4 °C and −5 °C suggests it acts as an early warning signal, leading to an increase in proline that prepares membrane and antioxidant systems before temperatures drop to lethal levels (−10 °C and −15 °C). Interestingly, SP_Zn+SA_HA treatment, which triggered peak gene expression particularly at 4 °C, showed its best physiological stability at this temperature but exhibited a sharp increase in TL and proline at −15 °C. This suggests that this method may lead to metabolic exhaustion while providing an intense acute response. Under chronic and excessive stress, better results were obtained compared to the more balanced and sustainable protection offered by SA_HA+Zn. Consequently, physiological data served as a functional validation of molecular profiles. The combined application of HA and Zn not only increases transcript levels but also translates into a tangible survival strategy. By optimizing the proline-water content-turgor axis and protecting cell membranes, these applications enable oat plants to maintain cellular homeostasis and structural integrity at various stages of cold and freezing stress.

## Conclusions

The results of this study demonstrated that the responses of oat plants to low-temperature stress are strongly modified by HA and Zn application strategies. Our findings highlighted that the combined application of HA and Zn (SA_HA+Zn, SP_HA+SA_Zn, and SP_Zn+SA_HA) enhanced cold tolerance through a complex but coordinated physiological and molecular network. The data revealed a clear cold tolerance pathway where upregulation of the CBF gene expression serves as the primary molecular response, likely followed by proline accumulation. This sequence effectively maintains leaf relative water content and membrane integrity, stabilizing the plant’s turgor under thermal stress. While this study established a link between CBF-mediated signaling and osmotic regulation, the full defense mechanism also involves complex antioxidant activities and nutrient uptake dynamics. Ongoing analyses of the antioxidant enzyme system (including superoxide dismutase (SOD), catalase (CAT), glutathione reductase (GR), and ascorbate peroxidase (APX)) and changes in mineral element uptake patterns under these treatments are being conducted. These findings, which provide a more detailed perspective on intrinsic homeostatic mechanisms, will be presented in our upcoming reports. In conclusion, this research serves as a pioneering framework for the use of integrated HA and Zn applications to enhance the intrinsic defense pathways of oat plants against environmental stressors. The practical results of these findings are significant for field applications, as the synergistic use of HA and Zn offers a cost-effective and sustainable strategy for stabilizing oat yields in high-risk frost zones. By optimizing the application methods identified in this study, farmers can increase crop resilience during the early growth stages when low-temperature stress is most harmful. Future studies will also investigate alternative signaling pathways such as the COR (Cold-Sensitive) gene family and various biostimulant combinations to further improve the intrinsic tolerance mechanisms of plants to environmental stressors. In addition, long-term field trials under varying environmental conditions are necessary to assess the consistency of these physiological benefits throughout the entire crop cycle.

## Supplemental Information

10.7717/peerj.20927/supp-1 Supplemental Information 1 Raw data.

10.7717/peerj.20927/supp-2 Supplemental Information 2 MIQE checklist.

## Additional Information and Declarations

### Data Availability

The following information was supplied regarding data availability:

The raw measurements are available in the Supplemental File.
